# In vitro and in vivo human metabolism and pharmacokinetics of S‐ and R‐praziquantel

**DOI:** 10.1002/prp2.618

**Published:** 2020-07-23

**Authors:** Nyasha Nicole Kapungu, Xueqing Li, Charles Nhachi, Collen Masimirembwa, Roslyn Stella Thelingwani

**Affiliations:** ^1^ African Institute of Biomedical Science and Technology (AiBST) Harare Zimbabwe; ^2^ Department of Clinical Pharmacology University of Zimbabwe (UZ) Harare Zimbabwe; ^3^ Research and Early Development, Cardiovascular, Renal and Metabolism, BioPharmaceuticals R&D AstraZeneca Gothenburg Sweden

**Keywords:** cytochrome‐P450, enantioselectivity, IVIVE, praziquantel

## Abstract

Racemic praziquantel (PZQ) is the drug of choice for the treatment of schistosomiasis. R‐Praziquantel (R‐PZQ) has been shown as the therapeutic form, whereas S‐PZQ is less efficacious and responsible for the bitter taste of the tablet. This study aimed at investigating the metabolism of R‐ and S‐PZQ as this could have implications on efficacy and safety of racemate and R‐PZQ specific formulations under development. In vitro CYP reaction phenotyping assay using 10 recombinant CYP (rCYP) isoenzymes showed hepatic CYP1A2, 2C19, 2D6, 3A4, and 3A5 were the major enzymes involved in metabolism of PZQ. Enzyme kinetic studies were performed by substrate depletion and metabolite formation methods, by incubating PZQ and its R‐ or S‐enantiomers in human liver microsomes (HLM) and the rCYP enzymes. The effect of selective CYP inhibitors on PZQ metabolism was assessed in HLM. CYP1A2, 2C19, and 3A4 exhibited different catalytic activity toward PZQ, R‐ and S‐enantiomers. Metabolism of R‐PZQ was mainly catalyzed by CYP1A2 and CYP2C19, whereas metabolism of S‐PZQ was mainly by CYP2C19 and CYP3A4. Based on metabolic CL_int_ obtained through formation of hydroxylated metabolites, CYP3A4 was estimated to contribute 89.88% to metabolism of S‐PZQ using SIMCYP^®^ IVIVE prediction. Reanalysis of samples from a human PZQ‐ketoconazole (KTZ) drug‐drug interaction pharmacokinetic study confirmed these findings in that KTZ, a potent inhibitor of CYP3A, selectively increased area under the curve of S‐PZQ by 68% and that of R‐PZQ by just 9%. Knowledge of enantioselective metabolism will enable better understanding of variable efficacy of PZQ in patients and the R‐PZQ formulation under development.

**Abbreviations**
4‐OH PZQ: 4‐hydroxy praziquantel
AUC: area under curve
Cl_H_: hepatic clearance
Cl_int_: intrinsic clearance
CL_uH,int_: intrinsic hepatic clearance
*C*_max_: maximum plasma concentration
CYP: cytochrome P450
DDI: drug‐drug interactions
HLM: human liver microsomes
IVIVE: in vitro to in vivo extrapolation
*K*_el_: elimination rate constant
*K*_M_: Michaelis Menten constant
KTZ: ketoconazole
PK: pharmacokinetic
PZQ: praziquantel
*V*_max_: maximum velocity
X‐OH PZQ: X‐hydroxy praziquantel

## 1 INTRODUCTION

Schistosomiasis is the second most important parasitic infection in the world after malaria with an estimated 250 million people being infected and approximately 800 million people at risk of infection.^1^ Schistosomiasis or Bilharzia is caused by schistosomes, which are parasitic trematode worms of the genus *Schistosoma*. Two thirds of the cases of schistosomiasis in sub‐Saharan Africa are attributed to *S haematobium* infection which causes severe urinary tract diseases^2^ and is a significant cause of clinical morbidity and disability. *Schistosoma mansoni* is also common in Africa^3^ while *Schistosoma japonicum* is confined to Asia.^4^ Disease control relies on chemotherapy, mass drug administration, and vector control especially in endemic regions. Praziquantel (PZQ) is the drug of choice in the treatment of schistosomiasis, based on its safety profile and effectiveness across all schistosome species.

Current formulations of PZQ are racemates consisting of *R*‐ and *S*‐enantiomers. *R*‐PZQ showed superior properties than its *S*‐enantiomer in biological activities^5^ and cytotoxicity,^6^ with much higher antischistosomal activity and worm reduction rates as indicated by much lower values in IC50 (approx. 500‐fold lower) and ED50 (5‐fold lower) against worms than its *S*‐enantiomer, as well as lower cytotoxicity in several cell lines. However, pharmacokinetics studies of PZQ showed that *R*‐PZQ is cleared 2.5 times faster than its *S*‐antipode from the circulating system with a maximum plasma concentration (*C*_max_) levels of only one third of *S*‐PZQ.^7^ PZQ is metabolized extensively in the liver to the main metabolite, 4‐hydroxy praziquantel (4‐OH PZQ)^8^ and numerous other mono‐, di‐, and tri‐hydroxyl metabolites and conjugates.^9^ The CYP3A4, CYP1A2, CYP2C9, and CYP2C19 have been identified as the main enzymes metabolizing the drug.^10^, ^11^ The plasma half‐life of PZQ is estimated to be between 1 and 3 hours^12^ and more than 80% of the drug is excreted within 24 hours in man.^13^ The systemic bioavailability of PZQ is, therefore, very low at <20%.^14^

In vivo, PZQ is well absorbed. More than 80% of the oral dose (taking into account unchanged drug and metabolites) is eliminated renally indicating a high fraction absorbed.^13^ PZQ demonstrates rapid first pass metabolism and high interindividual variability.^7^ The drug, however, relies on metabolism for excretion with <0.02% of unmetabolized PZQ being detected in urine.^15^ Therefore, any modulation of enzymes metabolizing PZQ is likely to have a major impact on the pharmacokinetics of the drug. PZQ metabolism is also affected by drug‐drug interactions (DDI) caused by either inhibition or induction of CYPs 1A2, 2C9, 2C19, and 3A4. For example, co‐administration of PZQ with cimetidine causes a 100% increase in its bioavailability^16^ most likely through the inhibition of CYP 1A2.

Other examples include inhibitory effects of grapefruit juice^17^ and ketoconazole (KTZ) on CYP3A4.^18^, ^19^ Co‐administration with food has also been shown to increase bioavailability. Induction has also been shown with rifampicin (CYP2C9, CYP2C19, and CYP3A)^20^ and dexamethasone (CYP3A4).^21^ Plasma PZQ concentrations decreased to undetectable levels in patients who had been pretreated with rifampicin for 5 days. A 50% reduction in PZQ plasma concentration was observed in patients with parenchymal brain cysticercosis who had been treated with dexamethasone a few days earlier to prevent PZQ related anti‐inflammatory side effects.

It was reported that CYP3A, CYP2C9, and CYP2C19 exhibited different catalytic activity toward PZQ enantiomers in vitro to PZQ metabolites observed in vivo in mice,^11^ however, there are no studies which have determined enantiospecific metabolism of R‐ and S‐PZQ in humans. In this study, we investigated stereoselective metabolism of PZQ in vitro and in vivo in man, to better understand and rationalize the mechanism behind the variable pharmacokinetics and selective clearance of R‐ and S‐PZQ.

## 2 MATERIALS AND METHODS

### 2.1 HLM and recombinant CYPs (rCYPs)

Recombinant human CYP (and human CYP‐reductase (NR) co‐expressed in *Escherichia coli*, 1A1 (CYP014), 1A2 (CYP001), 2A6 (CYP011), 2B6 (CYP020), 2C9 (CYP019), 2C19 (CYP008), 2D6 (CYP007), 2E1 (CYP009), 3A4 (CYP002), and 3A5 (CYP015) were obtained from CYPEX (CYPEX). Pooled human liver microsomes (HLM) (UltrapoolTM HLM150, equal gender mix, product number 452117) were obtained from BD Biosciences Genetest.

### 2.2 Human plasma samples

Plasma samples were obtained from the AiBST biobank. They were from a PZQ‐KTZ DDI pharmacokinetic study. In the study, the effect of KTZ (200 mg QD) on PZQ (20 mg/kg) was evaluated in a phase 1, open label, balanced, and randomized clinical trial involving 29 young male healthy volunteers.^19^ The study had a balanced, 2 sequence crossover designs with a wash out period of 7 days. Each participant served as their own control. Each participant provided written informed consent to take part in the study and the study was carried out according to Helsinki Declaration of 1975, as revised in 2008. The study was approved by the Medical Research Council of Zimbabwe (MRCZ) and the Medicines Controls Authority of Zimbabwe (MCAZ).

### 2.3 Materials

R‐PZQ, S‐PZQ, cis‐4‐hydroxy‐PZQ, and trans‐4‐hydroxy‐PZQ were a generous gift from Merck Serono. 5,5‐Diethyl‐1,3‐diphenyl‐2‐iminobarbituric acid was obtained from AstraZeneca. PZQ, sulfaphenazole, KTZ, quinidine, ticlopidine, furafylline, diazepam (DPZ), acetonitrile (ACN), β‐Nicotinamide adenine dinucleotide 2′‐phosphate reduced tetrasodium salt (NADPH), formic acid, potassium phosphate monobasic, and potassium phosphate dibasic were obtained from Sigma Chemical Co. All other reagents were of the highest obtainable grade.

### 2.4 In vitro incubations

#### 2.4.1 Qualitative substrate disappearance assay

All reactions were performed in triplicate in 96‐well plates. Each reaction mixture consisted of the appropriate enzyme (20 pmols rCYP or 0.5 mg/mL pooled HLM), substrate (1.0 µmol/L R‐, S‐ or racemic PZQ), 1 mmol/L NADPH, in 0.1 mol/L potassium phosphate buffer pH 7.4 in a final volume of 200 µL. The substrate was diluted to 40% ACN so that the percent organic component in the final solution was maintained at 1%. A final concentration of ACN of 1% has been shown to reduce the CYPEX rCYPs by <20%.^22^ Recombinant CYPs 1A1, 1A2, 2A6, 2B6, 2C8, 2C9, 2C19, 2D6, 3A4, and 3A5 were used. The reactions were initiated by addition of NADPH after a pre‐incubation of 5 minutes at 37°C. All reactions were terminated by the addition of 150 µL of ice‐cold ACN at either 0 minute (control) or 30 minutes (reaction). This was followed by centrifugation at 4500 *g* for 20 minutes. An aliquot (50 µL) supernatant was collected and 5 µL was injected and analyzed by liquid chromatography/tandem mass spectrometry (LC/MS/MS). Metabolism was signified qualitatively by loss of substrate from the media using the relationship:

$$\%\;\text{remaining compound}=1‐\frac{\text{Peak area at}\;T_{30\min}}{\text{Peak area at}\;T_{0\min}}\times 100.$$

#### 2.4.2 CYP selective diagnostic inhibition studies

Reactions were carried out in triplicate in 96 well plates. Each reaction was measured at 2 time points, that is at 0 and 30 minutes. Each reaction mixture consisted of the 0.5 mg/mL pooled HLM, substrate (1.0 µmol/L R‐, S‐ or racemic PZQ), inhibitor (10 µmol/L), and 0.1 mol/L potassium phosphate buffer pH 7.4 in a final volume of 200 µL. Inhibitor concentration of 10 µmol/L has been shown to be selective enough to show the contribution of some CYPs.^23^ The substrate was diluted to 40% ACN so that the percent organic component in the final solution was maintained at 1%. Furafylline, KTZ, ticlopidine, and quinidine were used as diagnostic inhibitors for CYP 1A2, 3A4/5, 2C19 and 2D6, respectively. The reactions were initiated by addition of NADPH after a pre‐incubation of 5 minutes at 37°C. All reactions were terminated by the addition of 150 µL ice‐cold ACN. Internal standard (0.2 µmol/L DPZ) was added to ACN prior to termination of the assay. This was followed by centrifugation at 4500 *g* for 20 minutes. An aliquot (50 µL) supernatant was collected and 5 µL was injected and analyzed by liquid chromatography/tandem mass spectrometry (LC/MS/MS). The percent effect of each inhibitor was the estimated contribution of the inhibited CYP calculated using the relationship below:

$$\begin{array}{c} \;\%\;\text{contribution}=\frac{\text{amount remaining with inhibitor}‐\text{amount remaining without inhibitor}}{\text{amount remaining without inhibitor}}\times 100. \end{array}$$

#### 2.4.3 Determination of intrinsic clearance

All reactions were performed in duplicate in reaction tubes. Generic incubation conditions with respect to substrate (R‐, S‐, or racemic PZQ) concentration (1 µmol/L), incubation time (60 minutes), NADPH concentration (1 mmol/L), and phosphate buffer (0.1 mol/L) were used. The generic conditions were previously described by Masimirembwa and co‐workers.^24^, ^25^ Each reaction mixture consisted of substrate incubated with appropriate enzyme, substrate, and NADPH in potassium phosphate buffer pH 7.4 in a final volume of 200 µL. The substrate was diluted to 40% ACN so that the percent organic component in the final solution was maintained at 1%. The reactions were quenched with 3 volumes of stop solution containing ACN and 0.8% formic acid at 0, 5, 10, 20, 30, 40, and 60 minutes. This was followed by centrifugation at 4500 *g* for 20 minutes. An aliquot (50 µL) supernatant was collected and diluted (1:1) in water prior to LC‐MS/MS analysis. The intrinsic clearance was calculated as follows:

$${\text{CL}}_{\text{int}}=1‐\frac{\ln2\times \text{incubation volume}\;}{T1/2\times \text{Protein or enzyme amount}}\times 100.$$

*T*_1/2_ was calculated from the elimination rate constant:

$$K=\frac{\ln2}{T1/2},$$

where CL_int_ is the intrinsic clearance, *T*1/2 is the half‐life of PZQ, incubation volume is the final volume of each reaction mixture and protein, or enzyme amount is the amount of protein in the reaction mixture. Each reaction was monitored over **7** time points.

#### 2.4.4 Enzyme kinetics

The reactions were carried out in duplicate at each time point. Incubation conditions used for the determination of *K*_M_ and *V*_max_ were optimized for linearity with respect to time and protein concentrations. The optimal linearity conditions for protein or enzyme concentration and incubation time established for the *K*_M_ and *V*_max_ studies were: 0.2 mg/mL and 15 minutes for HLM, 15 pmols/200 µL, and 15 minutes for CYP1A2, 8 pmol/200 µL and 15 minutes for CYP2C19, 10 pmols/200 µL and 10 minutes for CYP2D6, 3A4 and 3A5. The linearity conditions were optimized for racemic PZQ and the same conditions used for the 2 enantiomers. Incubations were conducted in duplicate at varying substrate concentrations (0, 2.3, 4.7, 9.4, 18.7, 37.5, 75, 100, 150, 200, and 300 µmol/L) and incubation were performed in a final volume of 200 µL as described in sections above. Metabolites were analyzed by LC‐MS/MS as described below. The *K*_M_ and *V*_max_ values of each marker substrate were determined using nonlinear least squares regression analysis in GraFit software (version 3.0, Erithacus Software Limited) and SigmaPlot Enzyme Kinetics Module for Windows 7.0 (SPSS, Inc).

#### 2.4.5 In vitro–in vivo extrapolation

In vitro metabolism data for R‐, S‐, and racemic PZQ with rCYPs were used for in vitro to in vivo extrapolation (IVIVE) using SIMCYP^®^ (version 17). The clearance of the incubation mixture was expressed as μL/min/pmol for rCYP.

Intrinsic Clearance can be scaled to the intrinsic hepatic clearance (CL_uH_,_int_ for the whole liver. In the scaling process the body weight for an average man was taken to be 80 kg, liver weight as 1680 g and MPPGL as 32 mg/g. The Inter System Extrapolation Factor (ISEF) was used to estimate the relative CYP abundance for each CYP to adjust for the calculation of percentage contribution of each enzyme. The ISEF values for CYP1A2, CYP2C19, CYP3A4/3A5, and CYP2D6 were given as 0.16, 0.2, 0.25, and 0.15, respectively, for the CYPEX high reductase system in SIMCYP^®^. Calculation of intrinsic hepatic clearance (CL_uH_,_int_) from in vitro substrate depletion data was done using the following Equation^26^ within the Simcyp^®^ program:

$$\begin{array}{c} {\text{CL}}_{\text{uH},\text{int}}\;=\sum_{j=1}^{m}\left(\frac{{\text{ISEF}}_{j}\times {\text{CL}}_{\text{int}}\times {\text{rCYP}}_{j}\times {\text{CYP}}_{j}\;\text{abundance}}{{\text{fumic}}_{j}}\times \text{MPPGL}\times \text{Liver weight}\right), \end{array}$$

where there are j CYPs with corresponding CL_int_ values calculated from enzyme kinetic parameters for the different pathways in each recombinant system, CL_int_ = in vitro clearance, MPPGL = microsomal protein per gram liver, ISEF*_j_* is the scaling factor for the corresponding CYP and fumic is fraction of drug unbound in microsomes which was predicted as 0.771. The CYP abundance for each of CYP enzymes used is given in Table A1.

*K*_M_ and *V*_max_ obtained from the enzyme kinetics assay were used to predict CL_int_ and subsequently hepatic clearance in the SIMCYP^®^ software under the enzyme kinetics module with X‐OH PZQ formed by CYP3A4, 4‐OH PZQ by CYP1A2 and CYP2C19 and Y‐OH PZQ by CYP2D6. The metabolite data such as physical chemical properties for 4‐OH‐PZQ were obtained from literature and that for X and Y‐OH PZQ were assumed based on the 4‐OH PZQ data. Calculation of intrinsic hepatic clearance (CL_uH_,_int_) from in vitro substrate depletion data was done using the following equation within the Simcyp^®^ program:

$$\begin{array}{c} \;{\text{CL}}_{\text{uH},\text{int}}=\sum_{j=1}^{m}\left(\frac{{\text{ISEF}}_{j}\times V_{\max}\left({\text{rhCYP}}_{j}\right)\times {\text{rCYP}}_{j}\;\text{abundance}}{K_{M}\left(\mu M\right)\left({\text{rhCYP}}_{j}\right)}\times \text{MPPGL}\times \text{Liver weight}\right), \end{array}$$

where *K*_M_ and *V*_max_ are obtained from the in vitro assay, ISEF*_j_* is the scaling factor for the corresponding CYP and MPPGL = microsomal protein per gram liver. The CYP abundance for each of CYP enzymes used is given in Table A1.

The well‐stirred model was used to estimate the hepatic clearance (CL_H_) due to metabolism. The model was chosen over the parallel tube and the dispersion model because of its simplicity and the fact that very small differences in predicted values by the 3 models have been observed.^27^ The hepatic clearance was expressed as^26^:

$${\text{CL}}_{H}=\frac{Q_{H}\times \text{fuB}\times {\text{CL}}_{\text{uH},\text{int}}}{Q_{H}+\left(\text{fuB}\times {\text{CL}}_{\text{uH},\text{int}}\right)},$$

where *Q*_H_ is the hepatic blood flow (1500 mL/min), CL_uH,int_ is clearance scaled to the whole liver, and reflects the actual metabolic capacity of the enzyme system and fuB is the free fraction of drug in blood calculated as fraction unbound in plasma (fu) divided by the blood to plasma drug concentration ratio (B/P). The value of fu used for PZQ, R‐PZQ, and S‐PZQ was 0.2^14^ and B/P for R and S PZQ was 0.8 and 0.78, respectively,^28^ obtained from studies reported in literature.

### 2.5 Analysis of R/S‐PZQ and hydroxylated metabolites using achiral UPLC‐MS/MS

R‐PZQ, S‐PZQ, 4‐OH PZQ, and X‐OH PZQ were analyzed by achiral UPLC/MS‐MS using a validated method as described in detail by Nleya and coworkers.^19^ Briefly, Chromatographic separation was achieved on an Acquity^®^ UPLC HSS T3 Column (2.1 × 30 mm, 1.8 μm, Waters) maintained a temperature of 40°C, using a gradient elution with a run time of 3 minutes. Mobile phase consisted of solvent A (0.2% formic acid and 2% ACN in water) and solvent B (0.2% formic acid in ACN) pumped at a flow rate of 0.8 mL/min as follows 96%‐30% A (0‐1.8 minutes), 5% A (2.0‐2.5 minutes), 96% A (2.6 minutes). Detection was done using a triple quadrupole Xevo TQS mass spectrometer (Waters) operating in ESI+ mode with Masslynx 4.1 running in MRM mode.

Four MRM transitions were followed simultaneously, PZQ (313.16 > 203.08), cis‐4‐OH‐PZQ (329.09 > 203.07), trans‐4‐OH‐PZQ (329.09 > 203.07 and 311.04 > 201.07), X‐OH‐PZQ (311.04 > 201.07), and 5,5‐Diethyl‐1,3‐diphenyl‐2‐iminobarbituric acid which was used as internal standard (336.36 > 195.5, dwell time = 0.025 seconds). The calibration curves were constructed within the range of 0.004‐2 μmol/L for PZQ and from 0.002 to 1 μmol/L for cis‐4‐OH‐PZQ and from 0.02 to 10 μmol/L for trans‐4‐OH‐PZQ. Quantitation was performed using Target Lynx (Waters). The concentrations of the analytes were calculated based on the peak area ratio of each compound to the internal standard and compared with the calibration curves. Due to the lack of reference compound for X‐OH‐PZQ, a semi‐quantitation of X‐OH‐PZQ was performed using the calibration curve of trans‐4‐OH‐PZQ. This was based on the similar fragmentation pattern and behavior in‐source. Trans‐OH‐PZQ undergoes extensive MS in‐source dehydration (loss of water), its dehydrated ion, m/z 311.

### 2.6 Determination of R‐ and S‐PZQ in human plasma samples

**Figure 1:**
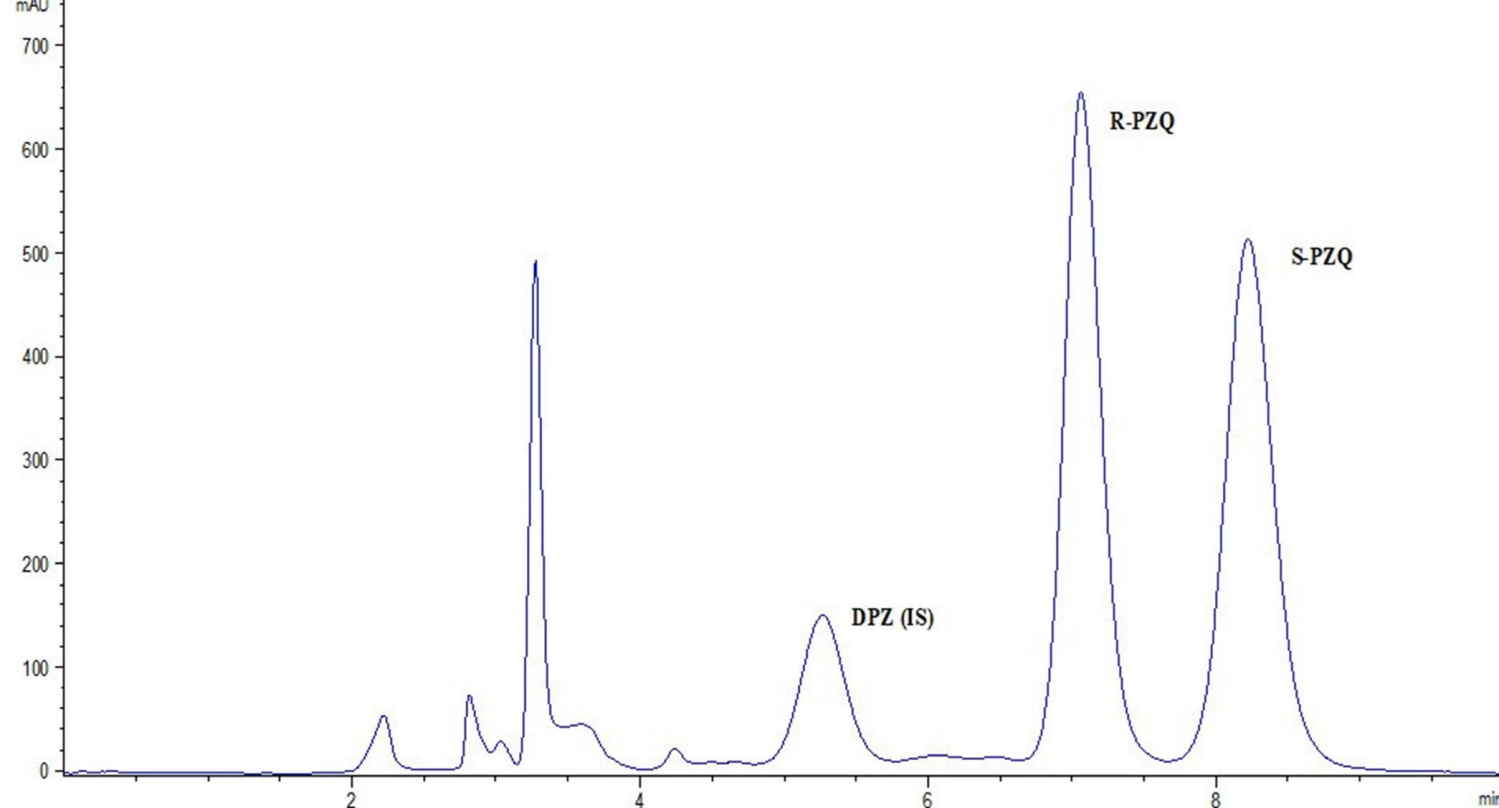
Representative chromatogram of R‐ and S‐PZQ in a plasma sample. The elution times for R‐PZQ, S‐PZQ, and diazepam (IS) were 7.1, 8.5, and 5.3 minutes

Protein precipitation was used to extract PZQ from human plasma samples from a previous study,^19^ Briefly, internal standard (10 µL DPZ) was added to 100 µL of plasma followed by extraction with 690 µL of ice‐cold ACN. The samples were vortexed for 30 seconds and left under gentle agitation for 25 minutes. The samples were then centrifuged at 11 200 rcf for 10 minutes. From the supernatant, 20 µL was injected onto the chiral HPLC system for analysis. Chiral chromatography was performed on an Agilent 1100 system with a quaternary pump delivering 0.1% formic acid 100% ACN at 0.6 mL/min. The column used for the chiral separation of R and S PZQ was an Astec Cellulose DMP (150 × 4.6, 5 µm) and the column temperature was kept at 15°C. Detection was done using a UV diode array detector at 210 nm. The retention times for R PZQ and S PZQ were 7.1 and 8.5 minutes, respectively, and that of IS was 5.3 minutes. The total run time was 10 minutes. A typical chromatogram of the chiral determination of R‐ and S‐PZQ in plasma is shown in Figure 1. The data were gathered and processed using Agilent OpenLab CDS for Chemstation software version 1.9. Linear calibration curves were plotted as concentration vs peak area ratio for analyte to IS with no weighting. R‐PZQ and S‐PZQ were linear in the range of 50‐10 000 ng/mL with an R value of 0.9997. The calibration curve was used to quantitate plasma PZQ concentrations.

### 2.7 Data analysis

The pharmacokinetic (PK) analysis was performed using the WinNonlin program, version 8.2 (Pharsight Corp). Mean PK profiles were plotted as graphs of R‐ or S‐PZQ as concentration vs time. A non‐compartmental approach was used for curve plotting and parameter calculation. The area under the curve (AUC) from time 0 to the last sampling time point (AUC*_t_*) was computed using the log‐linear trapezoidal rule. The linear trapezoidal rule was used for the ascending part of the PK curve, while the log trapezoidal rule was used for the descending part of the graph. The AUC extrapolated to infinite time was calculated as AUC_∞_ = AUC*_t_* + *C_t_*/*K*_el_ where *C_t_* is the concentration of the last sample taken and *K*_el_ is the terminal elimination rate constant. The terminal elimination rate constant *K*_el_ was determined by least squares regression analysis in the terminal phase of the semi log concentration‐time curve. The half‐life (*t*_½_) was calculated as *t*_½_ = 0.693/*K*_el_.

### 2.8 Statistical analysis

Statistical analysis was performed using the GraFit software (version 3.0, Erithacus Software Limited). Descriptive statistics, paired *t* tests, and the computation of 90% confidence intervals (CIs) were performed. The null hypothesis assumed no significant difference between the test and reference treatments. Analysis of variance (ANOVA) was performed on the AUC and *C*_max_ after transformation of the data to their natural logarithmic (ln) values. Using the error variance (S2) obtained from the ANOVA, the 90% CIs were calculated from the following equation:

$$90\%\;\text{CI}=\left({\bar{x}}_{T}‐{\bar{x}}_{R}\right)\pm t_{v}^{0.1}\cdot \sqrt{S^{2}\times \frac{2}{n}},$$

where ${\bar{x}}_{T}$ and ${\bar{x}}_{R}$ are the geometric means of the ln transformed values for the test treatment (T) and the reference treatment (*R*); *S* ^2^ is the error variance obtained from the ANOVA; *n* is the number of subjects, $t_{v}^{0.1}$ is the *t*‐value for 90% of the *t*‐distribution and *v* is the degree of freedom of the error variance from the ANOVA. The anti‐ln of the above CI values was then computed to give the 90% CIs of the ratios of the test to the reference treatment geometric means.

## 3 RESULTS

### 3.1 Qualitative substrate disappearance assay

**Figure 2:**
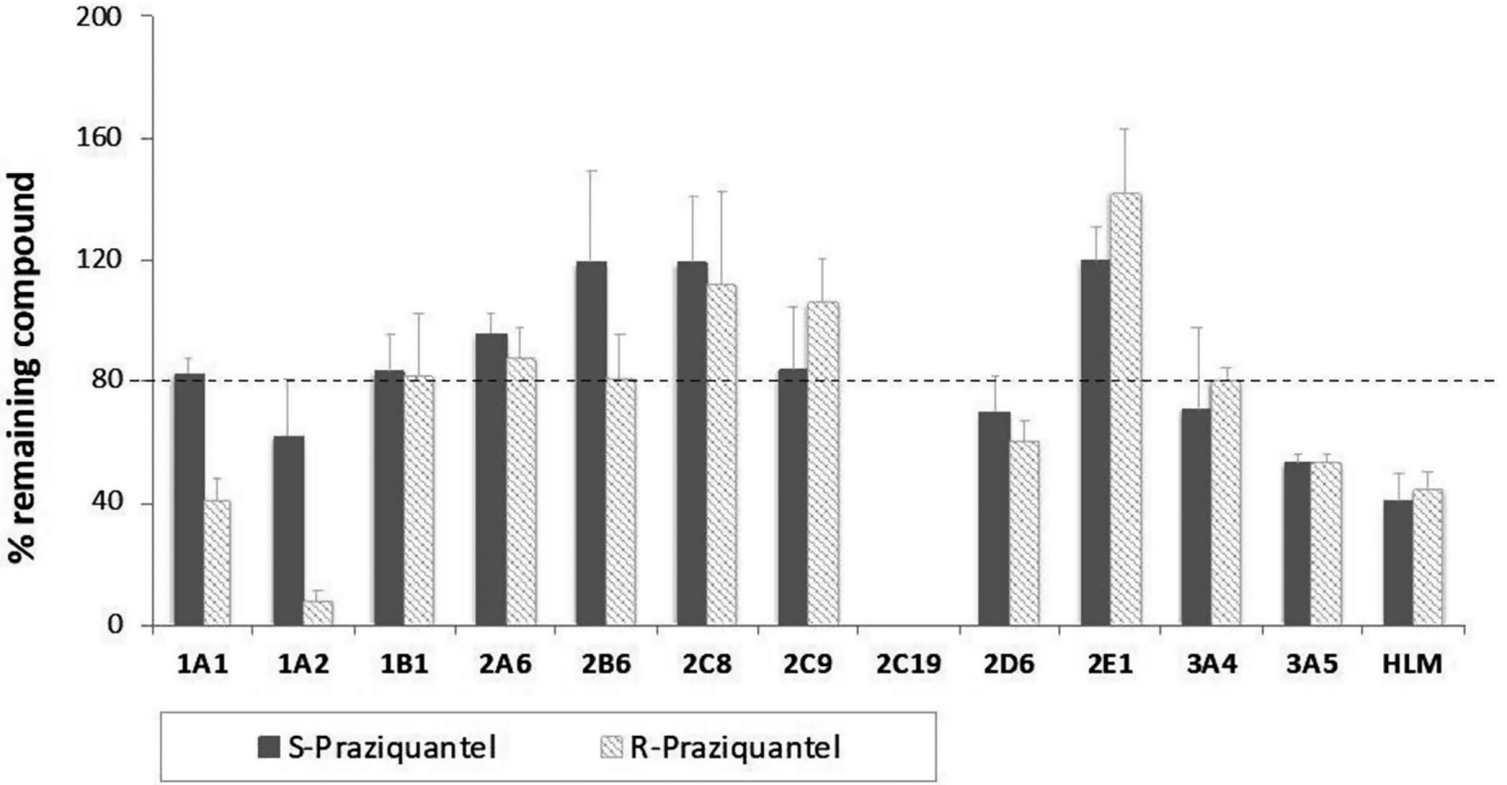
The disappearance of R‐ and S‐PZQ from media against a panel of drug metabolizing CYP enzymes

Percent depletion of substrate across a panel of rCYPs and HLM using the 2‐time point assay (0 and 30 minutes) identified the enzymes involved in the metabolism of R‐PZQ and S‐PZQ. The qualitative contribution of each enzyme is shown in Figure 2. Using a general cutoff of at least 20% depletion as representing significant metabolism, this screen assay showed that human rCYPs 1A1, 1A2, 2C19, 2D6, 3A4, and 3A5 were the enzymes involved in the metabolism of R‐PZQ and S‐PZQ with a complete depletion of R‐PZQ and S‐PZQ by CYP2C19. There was differential metabolism by CYP1A2 where R‐PZQ was depleted by approximately 5‐fold more than the S‐PZQ.

### 3.2 Inhibition by CYP selective inhibitors

The inhibition assay was performed in HLM to confirm the contribution of the identified CYP enzymes in the metabolism of PZQ and its individual enantiomers. The diagnostic inhibitors used, that is, furafylline (CYP1A2), ticlopidine (CYP2C19), quinidine (CYP2D6), and KTZ (3A4, 3A5) have been shown to be potent diagnostic inhibitors. Concentrations of PZQ and its enantiomers in HLM as well as the inhibitory effects of diagnostic inhibitors on metabolism are shown in Table 1. Due to the lack of high selectivity at the high inhibitor concentration, only relative contributions can be deduced from the results presented in Table 1. The greatest contribution to R‐PZQ metabolism was mainly by CYP2C19, CYP1A2, and CYP2D6 which had an almost equal contribution. The contribution of CYP3A4 was low for R‐PZQ. The inhibitory effects on CYP3A4, CYP2C19, and CYP1A2 activity by the selective inhibitors showed metabolic stereoselectivity. Metabolic stereoselectivity was observed with CYP1A2 where R‐PZQ was metabolized more as compared to S‐PZQ. CYP3A4 mainly contributes to S‐metabolism (Table 1). CY2C19 showed stereoselectivity by contributing more to the metabolism of S‐PZQ than of R‐PZQ. The activity was, however, lower for racemic PZQ for CYP1A2 as compared to R‐ and S‐PZQ.

**Table 1:** Relative contributions of the CYP1A2, CYP2C19, CYP3A4/5, and CYP2D6 to the metabolism of PZQ, R‐PZQ, and S‐PZQ in human liver microsomes (HLM) by inhibition studies to identify important contributing isoforms

PZQ	Blank^a^	Control (SD)^b^	CYP1A2 (SD)	CYP2D6 (SD)	CYP2C19 (SD)	CYP3A4/5 (SD)	Sum (%)
R/S	1 µmol/L	0.63 (0.18) µmol/L	0.43 (0.12) µmol/L (−54%)	0.76 (0.07) µmol/L (35%)	1.1 (0.12) µmol/L (127%)	0.99 (0.17) µmol/L (97%)	205
R	1 µmol/L	0.43 (0.01) µmol/L	0.78 (0.16) µmol/L (61%)	0.72 (0.07) µmol/L (51%)	0.79 (0.25) µmol/L (64%)	0.62 (0.03) µmol/L (33%)	209
S	1 µmol/L	0.48 (0.07) µmol/L	0.62 (0.15) µmol/L (27%)	0.76 (0.12) µmol/L (54%)	1.09 (0.04) µmol/L (117%)	1.16 (0.22) µmol/L (131%)	329

Note

Furafylline, quinidine, ticlopidine, and ketoconazole were used as diagnostic inhibitors of CYP1A2, CYP2D6, CYP2C19, and CYP3A4, respectively; the contribution of each isoform is shown in parenthesis.

^a^ Blank––Amount of PZQ before incubation.

^b^ Control**^––^**Amount of PZQ after incubation in the absence of an inhibitor.

### 3.3 Determination of intrinsic clearance (CL_int_) using the substrate depletion approach

The depletion rates of R‐ and S‐PZQ were determined in HLM and rCYPs 1A2, 2C19, 2D6, 3A4, and 3A5. The calculated intrinsic hepatic clearance and hepatic clearances are shown in Table 2. CYP2C19 was observed to have a high intrinsic clearance for both R‐ and S‐PZQ of 19.73 and 30.71 mL/min/kg. Clearance by CYP3A4 was 3 times faster for S‐PZQ as compared to R‐PZQ with predicted CL_uH,int_ of 7.46 and 2.50 mL/min/kg, respectively, whereas R‐PZQ was cleared 2 times faster by CYP1A2 as compared to S‐PZQ with predicted CL_uH,int_ of 27.80 and 13.07 mL/min/kg, respectively. The predicted CL_H_ for each isoenzyme showed enantioselective metabolism for R‐PZQ by CYP1A2 and S‐PZQ by CYP3A4 (Table 2). The predicted CL_H_ of R‐PZQ by CYP1A2 is 5.071 mL/min/kg vs that of S‐PZQ of 2.877 mL/min/kg and for CYP3A4 the CL_H_ values are 0.6048 and 1.759 mL/min/kg for R‐PZQ and S‐PZQ, respectively. No comparisons were made with the racemate since the concentration used was half for each enantiomer. No major differences were, however, expected since we did not observe any enantiomer‐enantiomer interaction. HLM‐ and CYP1A2‐mediated R‐PZQ was high. CYP3A4‐mediated R‐PZQ clearance was low, whereas the remainder of the compounds was medium clearance.

**Table 2:** Predicted intrinsic (CL_uH,int_) and hepatic clearance (CL_H_) of R‐ and S‐PZQ in HLM and recombinant CYPs

	*T*_1/2_ (min)		Predicted CL_uH,int_ (mL/min/kg)		Predicted CL_H_ (mL/min/kg)	
	R‐PZQ	S‐PZQ	R‐PZQ	S‐PZQ	R‐PZQ	S‐PZQ
HLM	11.46	29.50	172.0	97.50	13.06	10.78
CYP1A2	7.540	16.04	27.80	13.07	5.071	2.877
CYP2C19	3.600	4.130	19.73	30.71	3.904	5.600
CYP2D6	34.66	45.30	0.860	0.660	0.2132	0.1712
CYP3A4	130.8	29.12	2.500	7.460	0.6048	1.759
CYP3A5	42.52	35.01	3.750	4.680	0.8929	1.142

### 3.4 Enzyme kinetics of hydroxylated metabolites formation

The metabolism and formation of the main metabolites of racemic PZQ have been previously described.^10^ The formation of the 2 main in vitro metabolites namely cis‐4‐OH‐PZQ and X‐OH‐PZQ showed stereoselectivity. This was also reflected in the clearance (Table 3). The enzyme kinetics of the formation of the major hydroxylated metabolites, cis‐4‐OH‐PZQ and X‐OH‐PZQ, in HLM showed that the intrinsic clearance (CL_uH,int_) via the formation of X‐OH‐PZQ from racemic PZQ is 5 times more than that via the formation of 4‐OH‐PZQ (Table 3). The obtained CL_uH,int_ in rCYPs indicates the metabolic enantioselectivity of CYP1A2 for R‐PZQ than S‐PZQ with values of 7.55 and 0.83 mL/min/kg, respectively. Clearance by CYP2C19 with R‐PZQ was 2 times more than S‐PZQ with CL_uH,int_ of 4.60 and 2.44 mL/min/kg respectively. Clearance by CYP2D6 was low and did not show any significant difference between R‐ and S‐PZQ.

**Table 3:** Enzyme kinetic parameters for the metabolism of PZQ, R‐PZQ, and S‐PZQ to 4‐OH‐PZQ and X‐OH‐PZQ intrinsic hepatic (CL_uH,int_) and hepatic clearance (CL_H_) in HLM and recombinant CYPs 1A2, 2C19, 2D6, 3A4, and 3A5

	Parameters for X‐OH‐PZQ			Parameters for cis‐4‐OH‐PZQ			
	HLM	CYP3A4	CYP3A5	HLM	CYP1A2	CYP2C19	CYP2D6
R/S‐PZQ							
*K*_M_ (µmol/L) [SD]	118.6 [35.79]	37.30 [5.455]	40.40 [4.331]	32.60 [3.982]	10.80 [2.242]	4.700 [0.8368]	140.5
*V*_max_ (nmol/min/mg protein) [SD]	11.10 [2.074]	75.40 [4.956]	54.70 [2.702]	0.5978 [0.028]	9.80 [0.571]	8.500 [0.3387]	14.60
CL_u__H,__int_ (mL/min/kg)	80.00	62.82	31.56	15.63	6.870	4.600	0.1100
CL_H_ (mL/min/kg)	9.677	8.546	5.554	3.233	1.574	1.084	0.0272
R‐PZQ							
*K*_M_ (µmol/L) [SD]	29.30 [4.020]	21.90 [3.1463]	13.60 [2.558]	38.90 [5.469]	11.10 [3.504]	11.90 [1.770]	105.5
*V*_max_ (nmol/min/mg protein) [SD]	1.200 [0.06266]	31.60 [1.7262]	18.90 [1.359]	0.7572 [0.0447]	11.10 [0.9436]	18.30 [0.7856]	16.70
CL_uH,_ _int_ (mL/min/kg)	35.00	44.78	32.50	16.63	7.550	4.600	0.1700
CL_H_ (mL/min/kg)	5.966	7.009	5.669	3.402	1.716	1.084	0.0434
S‐PZQ							
*K*_M_ (µmol/L) [SD]	61.00 [22.12]	59.60 [9.781]	43.80 [6.690]	44.30 [12.06]	45.30 [15.92]	5.300 [0.7903]	131.7
*V*_max_ (nmol/min/mg protein) [SD]	6.300 [1.102]	105.2 [9.034]	72.00 [5.200]	0.2700 [0.02999]	5.000 [0.7459]	5.100 [0.1769]	7.500
CL_uH,int_ (mL/min/kg)	88.25	55.04	38.34	5.250	0.8300	2.440	0.0600
CL_H_ (mL/min/kg)	10.32	8.116	6.509	1.227	0.2136	0.6138	0.1610

**Figure 3:**
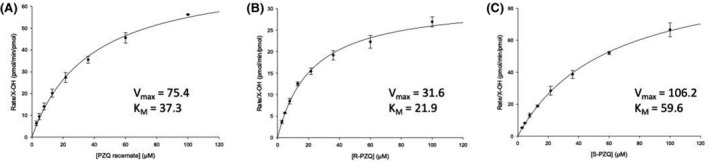
Michaelis‐Menten plot for the metabolism of (A) racemic‐PZQ, (B) R‐PZQ, and (C) S‐PZQ by CYP 3A4

There was higher affinity (low *K*_M_) for racemic PZQ and both enantiomers with CYP1A2 and CYP2C19 compared to CYP3A4 which showed low affinity as indicated by the higher *K*_M_ values. Although CYP2D6 contributed to metabolism of PZQ and its enantiomers, it had very low affinity characterized by a *K*_M_ greater than 100μM. The formation of X‐OH‐PZQ is mainly attributed to S‐PZQ metabolism rather than R‐PZQ metabolism with a *V*_max_ of 6.3 nmol/min/mg protein for S‐PZQ vs 1.2 nmol/min/mg protein for R‐PZQ. The Michaelis‐Menten kinetic plots for PZQ, R‐PZQ, and S‐PZQ metabolism by rCYP3A4 for the formation of X‐OH PZQ are shown in Figure 3. The high *V*_max_ for CYP3A4 with S‐PZQ shows that CYP3A4 metabolizes S‐PZQ with a higher turnover rate than R‐PZQ. This data agreed with our observations from the substrate disappearance assay (Figure 2) and previously determined clearance data (Table 2) as well as the results obtained from the inhibition assays (Table 1).

### 3.5 IVIVE using SIMCYP^®^

The relative percentage contribution of each of the enzymes to PZQ, R‐PZQ, and S‐PZQ metabolism was simulated using SIMCYP^®^ based on the in vitro metabolic Cl_int_ with rCYPs using substrate depletion method as well as the metabolite formation assays. From the substrate depletion assay, S‐PZQ was predicted to be mainly metabolized by CYP1A2, CYP2C19, and CYP3A4 (Table 4).The metabolite formation assay indicated CYP3A4 as the major contributor to the metabolism of all the 3 substrates (Table 4) which was not in agreement with substrate disappearance. The main contributors to the metabolism of R‐PZQ were CYP2C19 and CYP1A2 using the substrate depletion approach.

**Table 4:** Simcyp^®^ prediction of the relative percentage contribution of CYPs 1A2, 2C19, 3A4, 3A5, and 2D6 to R‐ and S‐PZQ metabolism using the metabolite formation and substrate depletion approach

	Percent contribution				
	CYP1A2	CYP2C19	CYP2D6	CYP3A4	CYP3A5
Substrate depletion					
R/S‐PZQ	35.71	46.12	2.030	13.42	2.720
R PZQ	67.24	19.77	2.870	8.080	2.050
S PZQ	38.31	31.14	2.480	25.56	2.510
Metabolite formation					
R/S‐PZQ	9.38	2.270	0.1900	82.78	5.380
R PZQ	13.24	2.520	0.3600	77.31	6.570
S PZQ	1.490	1.520	0.1300	89.88	6.980

**Figure 4:**
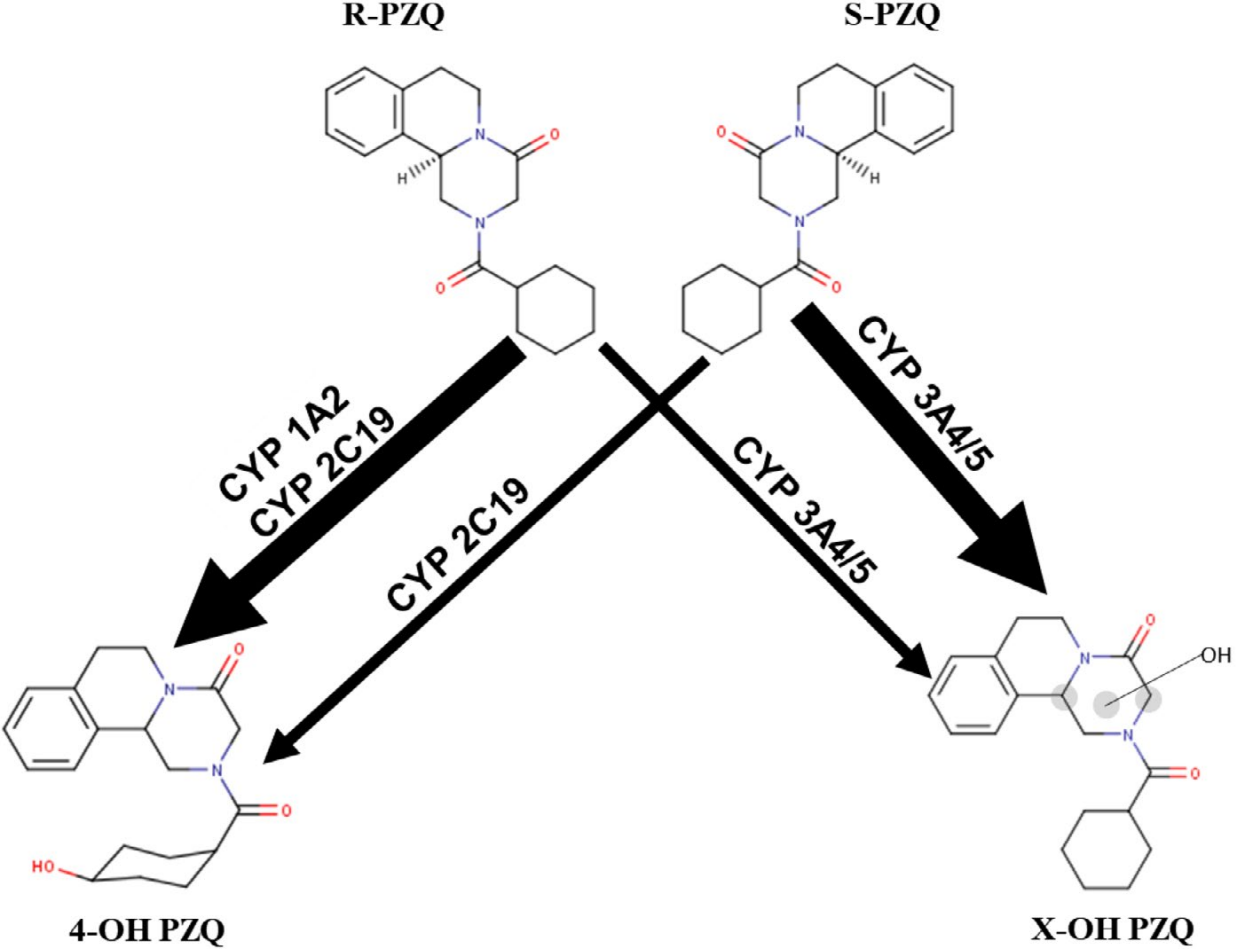
Postulated biotransformation pathways for R‐ and S‐PZQ to X‐OH‐PZQ and cis‐4‐OH‐PZQ based on in vitro and in vivo metabolism data

Predictions of CL_H_ for PZQ, R‐PZQ, and S‐PZQ using the metabolite formation and substrate depletion approaches were also conducted in SIMCYP^®^. The predicted total CL_H_ for PZQ, R‐PZQ, and S‐PZQ were 3.656, 6.923, and 6.265 mL/min/kg, respectively, using the substrate depletion assay and 11.44, 9.777, and 10.144 mL/min/kg, respectively, using the metabolite formation assay. The calculated hepatic clearance for PZQ, R‐PZQ, and S‐PZQ using the substrate depletion method was 11.70, 13.06, and 10.78 mL/min/kg, respectively, indicating that R‐PZQ is cleared faster than both PZQ and S‐PZQ. Predicted CL_H_ was indicative of intermediate clearance compounds as they exceed 3 quarters of hepatic blood flow rate. A depiction of the relative contribution of the enzymes to R‐PZQ and S‐PZQ metabolism using metabolite formation and substrate depletion approaches is shown in Figure 4. Due to circumstances we could not control, we could not establish our own inhouse ISEF values for the rCYP‐pooled HLM pairs, thus used the ISEF values for these rCYP batch with pooled HLM from CYPex that are in the SIMCYP simulator. This could have an effect on the accuracy of our predictions.

### 3.6 Impact of KTZ to pharmacokinetics of R‐ and S‐PZQ in human

Following oral administration of racemic PZQ to healthy volunteers, plasma samples were analyzed by chiral HPLC coupled with UV detection and the plasma concentrations of R‐ and S‐PZQ were determined. The relative bioavailability of R‐PZQ did not significantly change (*P* > .05) between the test (PZQ alone) and the treatment (PZQ + KTZ) with an AUC of 1346 ± 1470 ng/mL/h vs 1470 ± 767 (Table 5). The difference was 9.26%. There was, however, a 67.71% increase in bioavailability for S‐PZQ (3853 ± 1433 ng/mL/h vs 6462.98 ± 3102 ng/mL/h, *P* < .05). The mean *C*_max_ showed a decrease of 11.85% (313 ± 131 ng/mL vs 276 ± 89 ng/mL, *P* > .05) for R‐PZQ as compared to an increase of 107.11 (782 ± 171 ng/mL vs 1621 ± 212 ng/mL, *P* < .001) for S‐PZQ. The *C*_max_ of R‐PZQ was, therefore, 2.5 times less than that of S‐PZQ. The mean clearance (CL/F) was higher for R‐PZQ as compared to S‐PZQ (902 ± 264 L/h vs 340.08 ± 96.8 L/h). With the co‐administration of KTZ, the clearance of S‐PZQ reduced by a third (340 ± 97 L/h vs 211.51 ± 74 L/h, *P* < .05), whereas the clearance of R‐PZQ slightly increased (902.09 ± 264 L/h vs 1145.6 ± 992 L/h, *P* > .05).

**Table 5:** Pharmacokinetic parameters of R and S‐PZQ after single oral administration of 20 mg/kg PZQ (reference) followed by PZQ plus a fixed dose of 200 mg Ketoconazole (treatment) (n = 9)

PK parameter	Reference mean (SD) [geo mean]	Treatment mean (SD) [geo mean]	Geo mean ratio (T/R) % [90% CI]	Paired *t* test		
				Difference observed %	Significance of *p* value	Computed *p* value
R‐Praziquantel						
AUC_inf_ (h × ng/mL)	1345 (366.6) [1299]	1470 (766.6) [1288]	99.17 [65.25‐150.7]	9.290	>.05	.3132
${\text{AUC}}_{t_{0}‐t_{\text{last}}}$(h × ng/mL)	1244 (533.7) [1122]	1061 (539) [945.4]	84.27 [54.99‐129.1]	−14.68	>.05	.1541
*C*_max_ (ng/mL)	313.1 (131.1) [291.0]	276.0 (89.21) [263.0]	90.36 [66.60‐122.6]	−11.85	>.05	.1549
*T*_max_ (h)	3.000 (1.860) [2.590]	1.610 (0.600) [1.530]	—	−46.33	<.05	.0424
Half life (h)	3.340 (1.000) [3.210]	6.000 (6.520) [4.140]	—	79.64	>.05	0.1559
Clearance/F (L/h)	902.1 (264.9) [876.2]	1146 (992.1) [918.1]	—	26.99	>.05	.0913
*V*_d_/*F* (L)	4506 (2480) [4052]	7879 (7691) [5480]	—	74.87	>.05	.1431
Elimination rate constant (h^−1^)	0.2300 (0.07000) [0.2200]	0.2200 (0.1800) [0.1600]	—	−4.350	>.05	.5000
S‐Praziquantel						
AUC_inf_ (h × ng/mL)	3854 (1434) [3614]	6463 (3102) [5956]	164.8 [113.8‐238.7]	67.71	<.05	.0229
${\text{AUC}}_{t_{0}‐t_{\text{last}}}$ (h × ng/mL)	3147 (1169) [2973]	4912 (1663) [4682]	157.5 [119.2‐208.0]	56.09	<.001	.0006
*C*_max_ (ng/mL)	782.9 (171.7) [767.2]	1621 (212.7) [1610]	209.8 [181.6‐242.3]	107.1	<.001	.0000004
*T*_max_ (h)	3.000 (0.5000) [2.960]	2.440 (0.6800) [2.350]	—	22.95	<.05	.0106
Half life (h)	2.760 (1.770) [2.300]	3.630 (1.990) [3.170]	—	31.52	>.05	.1875
Clearance/F (L/h)	340.1 (96.37) [328.7]	211.5 (74.47) [200.3]	—	−37.81	<.05	.0083
*V*_d_/*F* (L)	1204 (552.6) [1092]	966.7 (339.0) [914.8]	—	19.73	>.05	.1842
Elimination rate constant (h^−1^)	0.3600 (0.2500) [0.300]	0.2500 (0.1500) [0.2200]	—	67.71	<.05	.0229

Note

Statistical calculations for AUC, and *C*_max_ were based on ln‐transformed data. Bioequivalence criteria are defined as 90% CI of the geometric mean ratios of T/R of between 80.0% and 125.0% for AUC_inf_, and *C*_max_. A single tailed, paired student *t*‐test was used to test for the differences between the means of the critical PK parameters: AUC, *C*_max_, *T*_max_, clearance, elimination rate constant (*K*_el_), and the apparent volume of distribution. The significance level was set at α = 0.05.

Abbreviations: AUC_inf_, AUC from time zero to infinity; ${\text{AUC}}_{t_{0}‐t_{\text{last}}}$ area under the plasma concentration–time curve from time zero to the last sampled time point; CI, confidence interval; *C*_max_, peak plasma concentration of the drug; SD, standard deviation; *T*_max_, time needed to achieve *C*_max_.

The FDA no effect boundary for clinical DDI studies for the 90% CI of the geometric mean ratio ranges between 80% and 125%.^29^ The reference and the treatment were significantly different for R‐PZQ by assessment of the 90% CI of the ratio of the geometric means of the log transformed AUC and *C*_max_. The computed values were 65.25%‐150.7% for AUC and 66.60%‐122.6% for the *C*_max_. The observation for S‐PZQ showed that the 2 treatments were significantly different where the 90% CI was 113.8%‐238.7% for AUC and 181.6%‐242.3% for *C*_max_. The values for the 90% CI showed that there was a greater effect on S‐PZQ than R‐PZQ with KTZ treatment.

## 4 DISCUSSION

Praziquantel has been shown to be metabolized mainly by cytochrome P450 enzymes in the liver, namely CYP 1A2, CYP2C19, CYP2C9, CYP 3A4, and CYP2D6.^10^, ^11^ Previous pharmacokinetic studies have shown R‐PZQ to have higher clearance than S‐PZQ.^7^, ^8^ Studies had earlier shown metabolism to be enantiomer selective,^7^, ^28^ but identification of the enzymes involved in R‐ and S‐PZQ metabolism had not been fully explored. Data from this study, therefore, are expected to contribute in providing an understanding of how best patient safety and efficacy can be maintained in the use of PZQ or its enantiomers. This is important in the development of PZQ formulations which contain 1 enantiomer especially when it has to be co‐administered with a drug which inhibit the enzymes required for metabolism. It also has implications on potential inducers of the drug of interest as this leads to subtherapeutic levels of PZQ.

Our reaction phenotyping study identified CYP1A1, CYP1A2, CYP2C19, CYP2D6, CYP3A4, and CYP3A5 as significant players in the metabolism of PZQ and its enantiomers (Figure 2). This agreed with our previous finding for PZQ metabolism^10^ and findings by Wang and co‐workers.^11^ There was, however, a discrepancy on CYP2C9 where our assay did not pick it as a major enzyme. The same was observed by Li and co‐workers.^10^ The Wang study used the metabolite formation approach. Although CYP2C9 showed to be an important contributor of the formation of metabolites that had been identified in mice, it could be contributing to a minor metabolic route in humans hence not resulting in significant substrate depletion of PZQ as observed in this study.

Clearance is an important parameter, a drug is termed a high clearance drug if the hepatic clearance (CL_H_) exceeds 14 mL/min/kg.^24^ The difference in CL_H_ between R‐PZQ and S‐PZQ is not as large as observed in vivo where R‐PZQ's clearance is 3 times faster than that of S‐PZQ. This could be attributed to the possible involvement of the intestinal metabolism where the CYPs are represented at different proportionalities than the liver.^7^, ^29^ The predicted total clearance (CL_H_) in Simcyp^®^ for PZQ, R‐PZQ, and S‐PZQ was 3.656, 6.923, and 6.265 mL/min/kg, respectively, using the substrate depletion assay and 11.44, 9.777, and 10.144 mL/min/kg, respectively, using the metabolite formation assay. The predicted CL_H_ gives PZQ via the substrate depletion assay as a low clearance drug which does not agree with 16 mL/min/kg reported by Li et.al.^10^ Analysis of IVIVE methods has shown that highly protein bound drugs tend to be underpredicted^30^ and there has not been a consensus about the importance of using bound or unbound drug as the object of clearance.^31^

To further investigate the contribution of CYP 1A2, CYP2C19, CYP 3A4/5, and CYP 2D6 to metabolism of PZQ, R‐PZQ, and S‐PZQ, the SIMCYP simulator was used. The contribution of the tested CYP isoforms to metabolism of R‐ and S‐PZQ metabolism showed CYP1A2 and CYP2C19 to be the main contributors of CYP metabolism using the substrate disappearance assay and the selective inhibition assay where the concentration of the isoforms were 1 μmol/L (Tables 1 and 2). There was significant enantiomer selectivity by CYP1A2 for R‐PZQ and CYP3A4/5 for S‐PZQ with CYP2C19 contributing to both R and S metabolism (Table 1). The sum of the total contribution by all isoforms was over 200% which could indicate lack of selectivity for the isoforms by the diagnostic inhibitors.^32^

However, in the predictions where data were obtained based on metabolite formation based on varying substrate concentration (Michaelis‐Menten Kinetics) the role of CYP3A4 became predominant (Table 3). Both CYP1A2 and CYP2C19 are high affinity enzymes and their role was important at the low concentration. However, as the concentration increased the role of low affinity CYP3A became more dominant. CYP1A2 and CYP2C19 are high affinity but saturable, whereas CYP3A4 is low affinity but high capacity. The concentration of the enzyme, therefore, is important as it can influence the outcome and interpretation of data.^10^

CYP1A2 contributed mostly to R‐PZQ metabolism when using substrate depletion method. It is consistent with the DDI of PZQ with albendazole, a CYP1A2 inhibitor, which resulted in an increase in R‐PZQ exposure.^28^ CYP3A4 contributed the most to S‐PZQ metabolite when using metabolite formation method. This observation was consistent with the observed PK data in this study (Table 5). However, if using the metabolite formation method, CYP3A4 contribute mostly to R‐PZQ metabolism, which was not observed in this study in man. The major disadvantage of the metabolite formation approach is that some compounds are not metabolized by only 1 CYP enzyme hence prior knowledge of all metabolites being investigated and enzymes contributing to the overall metabolic fate is required to consider the different pathways involved in the metabolism of the compound to accurately predict in vivo contribution and CL_H_.^33^ The metabolism of PZQ and its enantiomers is still to be fully characterized. In such cases, the substrate depletion approach has been demonstrated to be more effective. Prediction from in vitro data with experimental conditions optimal for CYPS might have implications on accuracy of prediction as gut CYP 3A4 might play a role in the metabolism of PZQ.

This study has 2 main limitations which are a potential source of bias. One such limitation is that the protein levels in rCYP and HLM were not matched to determine the free levels of PZQ and this could influence interpretation of data. The amount of protein in rCYP systems is usually much lower than that of HLM systems which causes a difference in the extent of nonspecific microsomal binding of the drug. The differences in nonspecific microsomal binding between the 2 systems lead to different *K*_M_ values, and therefore intrinsic clearance.^34^ Another limitation of the study is that it did not use laboratory specific ISEF values and this could have an impact on the predictions using SIMCYP. ISEF values account for the differences between rCYP and HLM as well as population variability in the CYP abundance for the CYP enzymes being assessed.^34^ Use of default ISEF values in the SIMCYP^®^ software could result in the reduction of accuracy of prediction to in vivo setting. However, a general trend can be observed, and further investigations can be made based on the results from this study.

At an inhibitor concentration of 10 µmol/L for KTZ and ticlopidine some unselectivity is observed for some CYPs hence limiting quantitative use of the level of inhibition as an accurate measure of contribution of the inhibited enzyme in the metabolism of a test compound.^10^ Using lower and potentially selective concentration, however, is associated with much lower inhibition, also not predictive of the contribution of inhibited enzyme. The use of “selective” CYP inhibitor at the 10 µmol/L used is best interpreted as demonstrating the participation of the inhibited CYP in the metabolism of the test compound and not an absolute quantitative measure of its contribution.

We are still facing challenges to unequivocally establish the chemical structure of X‐OH‐PZQ. However, assuming a 1:1 analytical response of trans‐4‐OH‐PZQ and X‐OH‐PZQ, the enzyme kinetic data (Table 3) indicate that the major route of metabolism results in the formation of X‐OH PZQ.

Our previous study demonstrated the role of CYP3A4 and CYP2C19 in the metabolism of PZQ to X‐OH PZQ and the role of CYP1A2 and CYP2C19 in the metabolism of PZQ to 4‐OH PZQ.^10^ From this study IVIVE simulation using SIMCYP^®^ software (Table 4), R‐PZQ is metabolized mainly by CYP 1A2 to 4‐OH PZQ and S‐PZQ is mainly metabolized by CYP3A4 to X‐OH PZQ. The enzyme CYP2C19 is important in the metabolism of both enantiomers. In the study by Nleya et al where PZQ metabolism was inhibited by KTZ, the exposure of X‐OH PZQ was reduced by 57%, while cis‐4‐OH PZQ exposure increased by 57% and trans‐4‐OH PZQ increased by 67%.^19^ The decrease in formation of X‐OH PZQ upon inhibition with KTZ shows that CYP 3A4 is responsible for the metabolism of PZQ to X‐OH PZQ and not 4‐OH PZQ. However, there is an in vitro phenomenon we do not have an explanation for yet where cis 4‐OH PZQ is mainly observed compared to in vivo where trans 4‐OH PZQ is the main metabolite.^28^, ^35^ The study by NLeya et al shows that trans 4‐OH PZQ is 30 times more than cis 4‐OH PZQ. Based on the present study, the study from Li *et al* and Nleya *et al* the postulated metabolic routes are displayed in Figure 4. To assess the risk for DDI that affect drug efficacy, drugs that inhibit CYP1A2 and CYP2C19 should be considered.

**Figure 5:**
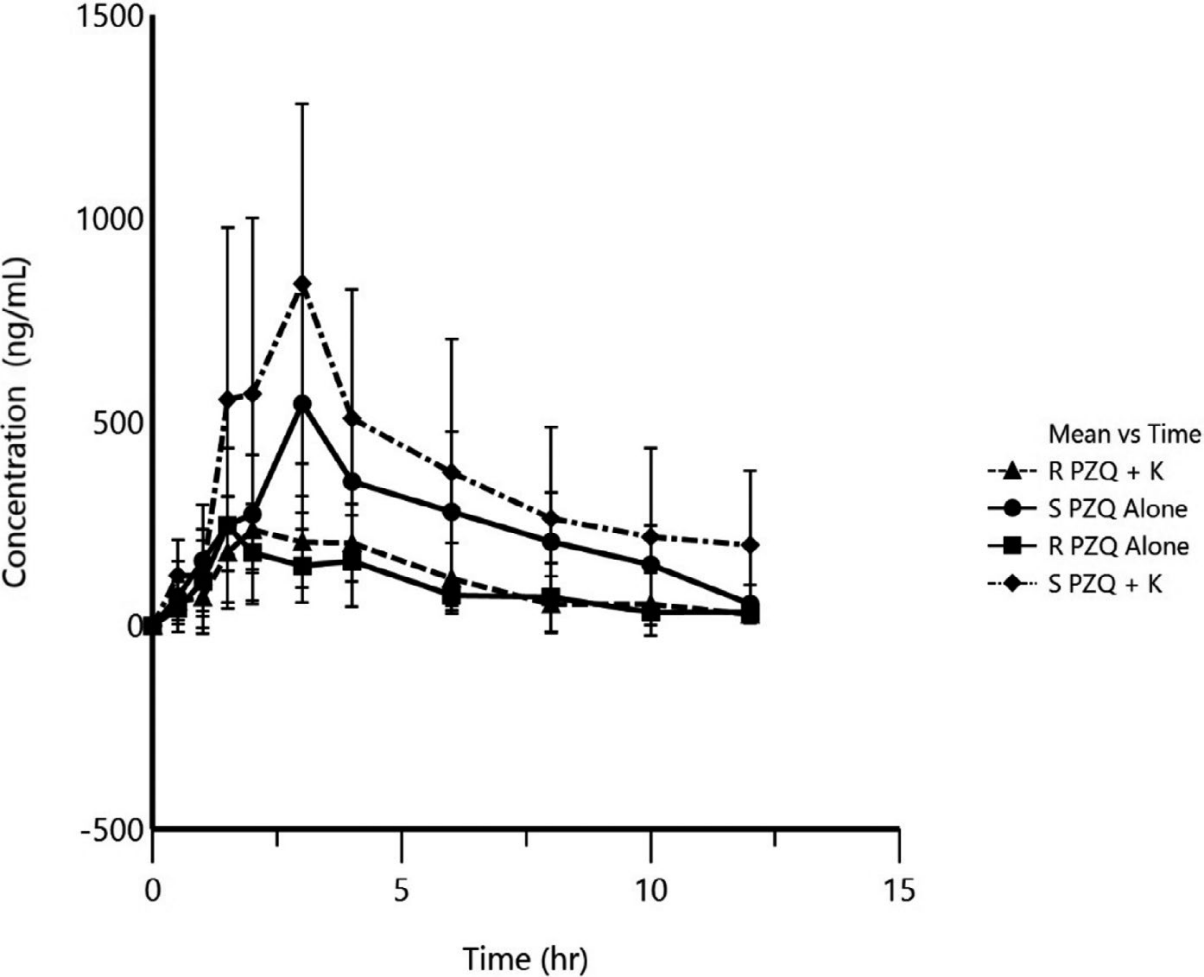
Mean Concentration vs time profile for R‐ and S‐PZQ following single dose administration of 40 mg/kg PZQ alone and together with 200 mg ketoconazole in healthy volunteers (n = 9)

The aspect of the in vitro R‐ and S‐PZQ enantiomer‐specific metabolism is supported by in vivo data on effect of KTZ, a potent CYP3A inhibitor, on the pharmacokinetics of S‐PZQ and R‐PZQ (Figure 5). However, with a limitation of lack of knowledge of the actual concentration of the drug that is presented to the drug metabolizing enzymes in the liver which might be different from the *C*_max_ observed in plasma and this might not reflect the absorbed drug entering the liver through the hepatic portal vein and the fraction unbound in plasma. The maximum plasma concentration (*C*_max_) and AUC obtained from the in vivo pharmacokinetic study was 0.16 µg/mL and 2.4 µg/mL × h for R‐PZQ and 0.54 µg/mL 3.3 µg/mL × h for S‐PZQ, respectively. The pharmacokinetic parameters obtained in our study are comparable to those reported in literature. The *C*_max_ is given as 0.16 µg/mL and 0.52 µg/mL for R‐ and S‐PZQ, respectively, from a previous study.^8^, ^28^ The bioavailability of R‐PZQ is considerably less than that of S‐PZQ. The co‐administration with the CYP3A4/5 inhibitor KTZ only increased the exposure of the ineffective S‐PZQ (68%) but not R‐PZQ (9%). Previous studies have observed that R‐PZQ is 100‐1000 times more potent in terms of anti‐schistosomal activity and hence therapeutic effect.^8^, ^33^ The increased exposure of S‐PZQ is, therefore, not likely to result in increased efficacy but rather a risk for increased toxicity. Caution should be taken when PZQ is dosed with CYP3A4 inhibitors. Although our in vivo study did not predict any significant DDI with R‐PZQ, values for S‐PZQ where, however, significant as indicated by the 90% of the geometric mean ratio which was outside the FDA 80‐125.

In this study we characterized the enantiomer selective metabolism of PZQ. R‐PZQ being mainly metabolized by CYP1A2 and CYP2C19, whereas S‐PZQ was mainly metabolized by CYP2C19 and CYP3A4. This finding adds to our knowledge of the potential metabolic basis of inter‐individual variation in R‐ and S‐PZQ exposure and resulting efficacy. It also provides a mechanistic basis of observed DDI when other drugs are co‐administered with PZQ and the possible implications of such DDI for PZQ efficacy and safety.

## 5 AUTHORSHIP CONTRIBUTIONS

*Participated in research design*: Collen Masimirembwa, Roslyn Thelingwani, Charles Nhachi. *Conducted experiments*: Roslyn Thelingwani, Xue‐qing Li, Nyasha Kapungu. *Performed data analysis*: Roslyn Thelingwani, Nyasha Kapungu. *Wrote or contributed to the writing of the manuscript*: Nyasha Kapungu, Xue‐qing Li, Charles Nhachi, Roslyn Thelingwani, Collen Masimirembwa.

## CONFLICT OF INTEREST

The authors declare that there is no conflict of interest.

## DATA SHARING AND DATA ACCESSIBILITY

The data that support the findings of this study are available on request from the corresponding author (RT). The data are not publicly available due to privacy or ethical restrictions.

## Supporting information

Table A1

## References

[1] Mo AX , Agosti JM , Walson JL , Hall BF , Gordon L . Schistosomiasis elimination strategies and potential role of a vaccine in achieving global health goals. Am J Trop Med Hyg. 2014;90(1):54‐60. 24402703 10.4269/ajtmh.13-0467 PMC3886428

[2] Olveda DU , Li Y , Olveda RM , et al. Bilharzia: pathology, diagnosis, management and control. Trop Med Surg. 2013;1(4):1‐19. 10.4172/2329-9088.1000135 PMC4208666 25346933

[3] Adenowo AF , Oyinloye BE , Ogunyinka BI , Kappo AP . Impact of human schistosomiasis in sub‐Saharan Africa. Braz J Infect Dis. 2015;19(2):196‐205. 25636189 10.1016/j.bjid.2014.11.004 PMC9425372

[4] Gordon C , Kurscheid J , Williams G , et al. Asian schistosomiasis: current status and prospects for control leading to elimination. Trop Med Infect Dis. 2019;4(1):40. 10.3390/tropicalmed4010040 PMC6473711 30813615

[5] Kovač J , Vargas M , Keiser J . In vitro and in vivo activity of R‐ and S‐ praziquantel enantiomers and the main human metabolite trans‐4‐hydroxy‐praziquantel against *Schistosoma haematobium* . Parasit Vectors. 2017;10(1):365. 28764732 10.1186/s13071-017-2293-3 PMC5540299

[6] Sun Q , Mao R , Wang D , Hu C , Zheng Y , Sun D . The cytotoxicity study of praziquantel enantiomers. Drug Des Devel Ther. 2016;10:2061‐2068. 10.2147/DDDT.S98096 PMC4928669 27445457

[7] Bustinduy AL , Waterhouse D , de Sousa‐Figueiredo JC , et al. Population pharmacokinetics and pharmacodynamics of praziquantel in Ugandan children with intestinal schistosomiasis: higher dosages are required for maximal efficacy. mBio. 2016;7(4):e00227–16. 27507822 10.1128/mBio.00227-16 PMC4992966

[8] Meister I , Ingram‐Sieber K , Cowan N , et al. Activity of praziquantel enantiomers and main metabolites against *Schistosoma mansoni* . Antimicrob Agents Chemother. 2014;58(9):5466‐5472. 24982093 10.1128/AAC.02741-14 PMC4135865

[9] Meier H , Blaschke G . Investigation of praziquantel metabolism in isolated rat hepatocytes. J Pharm Biomed Anal. 2001;26(3):409‐415. 11489386 10.1016/s0731-7085(01)00417-4

[10] Li XQ , Bjorkman A , Andersson TB , Gustafsson LL , Masimirembwa CM . Identification of human cytochrome P450s that metabolise anti‐parasitic drugs and predictions of in vivo drug hepatic clearance from in vitro data. Eur J Clin Pharmacol. 2003;59(5–6):429‐442. 12920490 10.1007/s00228-003-0636-9

[11] Wang H , Fang Z‐Z , Zheng Y , et al. Metabolic profiling of praziquantel enantiomers. Biochem Pharmacol. 2014;90(2):166‐178. 24821110 10.1016/j.bcp.2014.05.001 PMC4168258

[12] Leopold G , Ungethüm W , Groll E , Diekmann HW , Nowak H , Wegner DHG . Clinical pharmacology in normal volunteers of praziquantel, a new drug against schistosomes and cestodes ‐ an example of a complex study covering both tolerance and pharmacokinetics. Eur J Clin Pharmacol. 1978;14(4):281‐291. 729622 10.1007/BF00560463

[13] Patzschke K , Pütter J , Wegner LA , Horster FA , Diekmann HW . Serum concentrations and renal excretion in humans after oral administration of praziquantel‐‐results of three determination methods. Eur J Drug Metab Pharmacokinet. 1979;4(3):149‐156. Accessed August 1, 2017. 527600 10.1007/BF03189418

[14] González‐Esquivel D , Rivera J , Castro N , Yepez Mulia L , Jung CH . In vitro characterization of some biopharmaceutical properties of praziquantel. Int J Pharm. 2005;295(1–2):93‐99. 15847994 10.1016/j.ijpharm.2005.01.033

[15] Andrews P , Thomas H , Pohlke R , Seubert J . Praziquantel. Med Res Rev. 1983;3(2):147‐200. 6408323 10.1002/med.2610030204

[16] Jung H , Medina R , Castro N , Corona T , Nacional I , Neurologia D . Pharmacokinetic study of praziquantel administered alone and in combination with cimetidine in a single‐day therapeutic regimen. Antimicrob Agents Chemother. 1997;41(6):1256‐1259. 9174180 10.1128/aac.41.6.1256 PMC163896

[17] Castro N , Jung H , Medina R , González‐Esquivel D , Lopez M , Sotelo J . Interaction between grapefruit juice and praziquantel in humans. Antimicrob Agents Chemother. 2002;46(5):1614‐1616. 11959616 10.1128/AAC.46.5.1614-1616.2002 PMC127135

[18] Cioli D , Pica‐Mattoccia L . Praziquantel. Parasitol Res. 2003;90(supp 1):S3‐S9. 12811543 10.1007/s00436-002-0751-z

[19] Nleya L , Thelingwani R , Li X‐Q , et al. The effect of ketoconazole on praziquantel pharmacokinetics and the role of CYP3A4 in the formation of X‐OH‐praziquantel and not 4‐OH‐praziquantel. Eur J Clin Pharmacol. 2019;75:1077‐1087. 31089768 10.1007/s00228-019-02663-8

[20] Ridtitid W , Wongnawa M , Mahatthanatrakul W , Punyo J , Sunbhanich M . Rifampin markedly decreases plasma concentrations of praziquantel in healthy volunteers. Clin Pharmacol Ther. 2002;450:505‐513. 10.1067/mcp.2002.129319 12426514

[21] Vazquez ML , Jung H , Sotelo J . Plasma levels of praziquantel decrease when dexamethasone is given simultaneously. Neurology. 1987;37(9):1561‐1562. 3627459 10.1212/wnl.37.9.1561

[22] Baird J , Begg C , Pritchard MP , Voice MW . Effects of common organic solvents on the activity of recombinant human cytochrome P450s expressed in *E coli* . Issx/Jssx. 2005;(January 2007):450.

[23] Masimirembwa CM , Otter C , Berg M , et al. Heterologous expression and kinetic characterization of human cytochromes p‐450: validation of a pharmaceutical tool for drug metabolism research. Drug Metab Dispos. 1999;27(10):1117‐1122. Accessed April 30, 2020. 10497136

[24] Masimirembwa CM , Thompson R , Andersson TB . In vitro high throughput screening of compounds for favorable metabolic properties in drug discovery. Comb Chem High Throughput Screen. 2001;4:245‐263. 11375740 10.2174/1386207013331101

[25] Masimirembwa CM , Bredberg U , Andersson TB . Metabolic stability for drug discovery and development pharmacokinetic and biochemical challenges. Clin Pharmacokinet. 2003;42(6):515‐528. 12793837 10.2165/00003088-200342060-00002

[26] Shiran MR , Proctor NJ , Howgate EM , Rowland‐Yeo K , Tucker GT , Rostami‐Hodjegan A . Prediction of metabolic drug clearance in humans: In vitro‐in vivo extrapolation vs allometric scaling. Xenobiotica. 2006;36(7):567‐580. 16864504 10.1080/00498250600761662

[27] Houston BJ , Carlile DJ . Prediction of hepatic clearance from microsomes, hepatocytes, and liver slices. Drug Metab Rev. 1997;29(4):891‐922. 9421679 10.3109/03602539709002237

[28] Meister I , Kovac J , Duthaler U , et al. Pharmacokinetic study of praziquantel enantiomers and its main metabolite R‐trans‐4‐OH‐PZQ in plasma, blood and dried blood spots in *Opisthorchis viverrini*‐infected patients. PLoS Negl Trop Dis. 2016;10(5):e0004700. 27152952 10.1371/journal.pntd.0004700 PMC4859549

[29] Schuirmann DJ . A comparison of the two one‐sided tests procedure and the power approach for assessing the equivalence of average bioavailability. J Pharmacokinet Biopharm. 1987;15(6):657‐680. 3450848 10.1007/BF01068419

[30] Ring BJ , Chien JY , Adkison KK , et al. PhRMA CPCDC initiative on predictive models of human pharmacokinetics, part 3: comparative assessement of prediction methods of human clearance. J Pharm Sci. 2011;100(10):4090‐4110. 21541938 10.1002/jps.22552

[31] Hallifax D , Houston J . Methodological uncertainty in quantitative prediction of human hepatic clearance from in vitro experimental systems. Curr Drug Metab. 2009;10(3):307‐321. 19442091 10.2174/138920009787846341

[32] Khojasteh SC , Prabhu S , Kenny JR , Halladay JS , Lu AYH . Chemical inhibitors of cytochrome P450 isoforms in human liver microsomes : a re‐evaluation of P450 isoform selectivity. Eur J Drug Metab Pharmacokinet. 2011;36:1‐16. 21336516 10.1007/s13318-011-0024-2

[33] Jones HM , Houston JB . Substrate depletion approach for determining in vitro metabolic clearance: time dependencies in hepatocyte and microsomal incubations. Drug Metab Dispos. 2004;32(9):973‐982. 15319339 10.1124/dmd.104.000125

[34] Proctor NJ , Tucker GT , Rostami‐Hodjegan A . Predicting drug clearance from recombinantly expressed CYPs: intersystem extrapolation factors. Xenobiotica. 2004;34(2):151‐178. 14985145 10.1080/00498250310001646353

[35] Kovač J , Meister I , Neodo A , et al. Pharmacokinetics of praziquantel in *Schistosoma mansoni*‐ and *Schistosoma haematobium*‐infected school‐ and preschool‐aged children. Antimicrob Agents Chemother. 2018;62(8):e02253‐17. 29866859 10.1128/AAC.02253-17 PMC6105791

