# Supplementary material for: In vitro and in vivo human metabolism and pharmacokinetics of S‐ and R‐praziquantel
Source: Pharmacol Res Perspect. 2020 Jul 23;8(4):e00618. doi: 10.1002/prp2.618 (PMC7376644; doi:10.1002/prp2.618)
Supplement: Supplementary file 1 — Table A1 [file PRP2-8-e00618-s001.docx]

**Appendix**

Table1: SIMCYP Parameters for IVIVE and predicted Hepatic clearance (CL_H_) for each CYP isoform

| **Enzyme** | **CYP Abundance** | **ISEF Value (Cypex HR)** |
| --- | --- | --- |
| **CYP 1A2** | 52 | 0.16 |
| **CYP 2C19** | 14 | 0.20 |
| **CYP 3A4** | 137 | 0.25 |
| **CYP 3A5** | 103 | 0.25 |
| **CYP 2D6** | 8 | 0.15 |
